# Clinical Manifestation of Depression after Stroke: Is It Different from Depression in Other Patient Populations?

**DOI:** 10.1371/journal.pone.0144450

**Published:** 2015-12-04

**Authors:** Janneke M. de Man-van Ginkel, Thóra B. Hafsteinsdóttir, Eline Lindeman, Mirjam I. Geerlings, Diederick E. Grobbee, Marieke J. Schuurmans

**Affiliations:** 1 Department of Rehabilitation, Nursing Science and Sports, Brain Center Rudolf Magnus, University Medical Center Utrecht, Utrecht, The Netherlands; 2 Nursing Science, program in Clinical Health Sciences University Medical Center Utrecht, Utrecht, The Netherlands; 3 University of Professional Education Utrecht, Department of Healthcare, Utrecht, The Netherlands; 4 Faculty of Nursing, University of Iceland, Reykjavik, Iceland; 5 Centre of Excellence for Rehabilitation Medicine, Rehabilitation Center ‘De Hoogstraat’, The Netherlands; 6 Julius Center for Health Sciences and Primary Care, University Medical Center Utrecht, Utrecht, The Netherlands; Charité-Universitätsmedizin Berlin, GERMANY

## Abstract

**Background:**

Despite ample research on depression after stroke, the debate continues regarding whether symptoms such as sleep disturbances, loss of energy, changes in appetite and diminished concentration should be considered to be consequences of stroke or general symptoms of depression. By comparing symptoms in depressed and non-depressed stroke patients with patients in general practice and patients with symptomatic atherosclerotic diseases, we aim to further clarify similarities and distinctions of depression after stroke and depression in other patient populations. Based on this, it is possible to determine if somatic symptoms should be evaluated in stroke patients in diagnosing depression after stroke.

**Methods:**

An observational multicenter study is conducted in three hospitals and seven general practices including 382 stroke patients admitted to hospital with a clinical diagnosis of intracerebral hemorrhage or ischemic infarction, 1160 patients in general practice (PREDICT-NL), and 530 patients with symptomatic atherosclerotic diseases (SMART-Medea).

**Results:**

The prevalence of major depressive disorder according to DSM-IV criteria was 14.1% (95% CI 11.0%-18.0%) in the stroke cohort, 5.4% (95% CI 3.8%-7.9%) in the symptomatic atherosclerotic diseases cohort and 12.9% (95% CI 11.1%-15.0%) in the general practice cohorts. Comparing depressed patients of the three cohorts demonstrated broadly similar symptom profiles, as well as comparable levels of individual symptom prevalence. However, the stroke patients suffered more severely from these symptoms than patients in the other populations.

**Conclusions:**

The findings suggest that depression after stroke is not a different type of depression. This finding indicates that all depressive symptoms should be evaluated in stroke patients, including somatic symptoms.

## Introduction

Depression after stroke is a frequent complication that affects approximately one third of all patients in the first two years after the stroke [1,2]. It has been associated with increased functional, cognitive and communicative disability, reduced quality of life, and increased mortality [1,3–6]. Therefore, depression after stroke negatively impacts patient participation in rehabilitation and associated patient outcomes [2]. There is increasing evidence that treatment with antidepressants decreases the severity of depression [2] and improves functional status [5]. Therefore, the early detection of PSD is essential to optimize the recovery of stroke patients.

Despite the frequent occurrence and high impact of depression after stroke, questions still persist concerning its nature. There is an on-going debate in the literature regarding whether depression after stroke is caused by biological factors provoked by the brain injury and vascular pathophysiology underlying the stroke or whether it is a secondary psychological response to the physical, cognitive, and social impairments produced by the stroke itself [7,8]. The question remains whether somatic symptoms such as sleep disturbances, loss of energy, changes in appetite and diminished concentration should be considered to be clinical manifestations of depression after stroke or secondary consequences of the stroke [9].

Although many studies have been conducted on various aspects of depression after stroke, such as prevalence, screening measurements, associated factors, treatment and prevention [1–4,10–14], only a few studies have investigated the clinical manifestation of depression in stroke patients [15–26]. Moreover, meaningful comparison of the studies is hampered by the diverse methods and measurement instruments used to diagnose depression, the time elapsed between stroke onset and data collection, the patient groups compared with the stroke patients, and the data analysis. Hence, the question still remains whether the clinical manifestation of depression after stroke differs from that in other groups of patients. The present study was conducted to determine whether depressive symptom profiles are different in depressed stroke patients compared with non-depressed stroke patients, depressed patients with symptomatic atherosclerotic diseases other than stroke and depressed patients in general practice.

## Methods

### Study design and participants

A multicenter study was conducted in three hospitals in The Netherlands. Between December 2009 and January 2011, we included 410 consecutive stroke patients who were admitted to the hospitals with a clinical diagnosis of intracerebral hemorrhage or ischemic infarction in the first week after stroke onset who did not present with serious cognitive disorders as determined by the Mini Mental State Examination [27] (score ≥18) or communicative disorders based on the Frenchay Aphasia Screening Test (scores ≥17 for patients <60 years of age, ≥16 in patients ≥60, and ≥15 in patients ≥71 years of age [28].

For comparison, we used data on patients with symptomatic atherosclerotic diseases other than stroke (n = 592) from the Second Manifestation of Arterial Disease-Memory, depression, and aging study (SMART-Medea) [29], and data on patients in general practice from the PREDICT-NL study [30]. The SMART-Medea study is a prospective cohort study aimed at investigating brain changes on MRI associated with depression in patients with symptomatic coronary artery disease, cerebrovascular disease, peripheral arterial disease or an abdominal aortic aneurysm [29].

Predict-NL, the Dutch arm of the PredictD study, is a prospective cohort study that began in 2003. Consecutive general practice patients (n = 1338), recruited from seven general practices in the city of Utrecht and the surrounding areas, were approached to participate, irrespective of their reasons for consulting the general practitioner [30]. The design of the PredictD and the Predict-NL study was published previously [30,31]. For all studies, ethical approval was obtained from the Medical Ethical Committee of the University Medical Center Utrecht, and written informed consent was obtained from all participants.

### Outcome measures

Major depressive disorder was assessed according to the DSM-IV-TR criteria [32] using the Composite International Diagnostic Interview (CIDI) [33]. The CIDI is a structured diagnostic interview for DSM-IV psychiatric disorders that is designed to meet the need for a short but accurate, structured psychiatric interview for multi-center clinical trials and epidemiological studies [34]. The CIDI shows good diagnostic concordance with the DSM-III-R (К = 0.84) and the ICD-10 diagnosis (К = 0.78) for major depression [34]. Its reliability is good, with an inter-rater reliability К = 0.84, and a test-retest reliability of К = 0.90 [34]. The depression section of the CIDI-auto 2.1 version was administered by trained researchers.

The severity of depressive symptoms was measured with the 9-item Patient Health Questionnaire (PHQ-9) [35]. The items include the 9 symptoms of depression according to the DSM-IV-TR [32]. Patients rated how often they had been bothered by any of the symptoms during the previous two weeks using a 4-point Likert scale ranging from 0 (symptom not at all present) to 3 (symptom present nearly every day) [35]. Adding up the item scores yields a sum score ranging from 0 (no depressive symptoms) to 27 (all symptoms occurring nearly every day). The PHQ-9 was shown to perform well in medical settings [36], both in stroke patients [37] and general practice patients [38].

The stroke patients were visited at home or at the residential health care facility in the 6^th^-8^th^ week after stroke onset, and the CIDI and PHQ-9 were administered for the diagnosis of major depressive disorder since stroke onset and the prevalence of the depressive symptoms. In patients with symptomatic atherosclerotic diseases the PHQ-9 was administered in the same hospital visit. The general practice patients filled out the PHQ-9 at home and return the questionnaire by mail. In patients with symptomatic atherosclerotic diseases as well as general practice patients, the CIDI was administered during a hospital visit and general practice visit, respectively, to establish whether the patients had suffered from major depressive disorder during the past 6 months or 12 months, respectively. The researchers conducting the CIDI were blinded for the PHQ-9 answers.

### Data analysis

Prevalence rates for depression and individual symptoms were analyzed with descriptive statistical methods. Cases with missing data were excluded from the analyses. Of the 410 included patients in the stroke cohort, 382 (93.2%) patients remained after excluding cases with incomplete data regarding depression diagnosis or symptoms. In the symptomatic atherosclerotic diseases and general practice cohorts, data were complete in 530 (89.5%) and 1160 (86.7%) patients, respectively.

Demographic characteristics were compared between de depressed versus non-depressed patients of the stroke cohort and between the depressed patients of the stroke cohort versus the depressed patients of the other cohorts using percentages and Chi-square tests for dichotomous variables and either means with standard deviation (SD) and Student’s *t*-test or the median and interquartile range (IQR) with the Mann-Whitney *U*-test for continuous variables, depending on the normality.

To analyze differences in sum scores of the PHQ-9 among the cohorts, we assessed the normality of the data using the Kolmogorov Smirnov test and confirmed that the data were positively skewed. Therefore, we used the median scores with their interquartile ranges and employed the Mann-Whitney U-test in depressed versus non-depressed patients of the stroke cohort and the one-way ANOVA in depressed patients of the stroke cohort versus the other cohorts separately.

In addition, the answers to the PHQ-9 items were dichotomized at a threshold value of 2 indicating ‘symptom present more than half the days’ according to previous research [35]. Based on this threshold, the values 0 (not at all) and 1 (several days) were coded as ‘symptom present less than half the days’, and the answers 2 (more than half the days) and 3 (nearly every day) were coded as ‘symptom present at least half the days’. Then, the presence of each of the depressive symptoms was compared between the depressed patients in the stroke cohort and the other patient cohorts using Chi-square tests. Based on recently recommended cut-off scores [38], we also dichotomized the nine items into ‘symptom present at least at several days (values 1 [several days] to 3 [nearly every day]) or symptom absent (value 0 [not at all]). These analyses were repeated in the non-depressed patients of all the other cohorts. Finally, multivariable logistic regression was used to analyze whether age and sex biased the effects of the populations on the prevalence of the individual symptoms of depression.

## Results

The mean age of the 382 stroke patients was 69 years (SD = 14.5, range 20–97 years), and 207 (54.2%) of these patients were male. Of the patients with other symptomatic atherosclerotic diseases, the mean age was 61.6 years (SD = 9.61, range 31–83 years), and 431 (81.3%) were male; in the patients in general practice, the mean age was 50.5 years (SD = 16.3, range 18–88 years), and 438 (37.8%) were male. Table 1 show the baseline and clinical characteristics of patients included in the study.

**Table 1:** Demographic and clinical characteristics of the three cohorts.

Characteristics		Depressed patients based on the CIDI			Non-depressed patients based on the CIDI		
		Stroke *n* = 54	Symptomatic atherosclerotic disease other than stroke *n* = 29	General practice *n* = 150	Stroke *n* = 328	Symptomatic atherosclerotic disease other than stroke *n* = 501	General practice *n* = 1010
Sex n (%)							
	Male	26 (48.1)	24 (82.8)*	54 (36.0)*	181 (55.2)	407 (81.2)	382 (37.8)
	Unknown	- (-)	- (-)	- (-)	- (-)	6 (1.2)	- (-)
Age in years Mean (SD) (min-max)		65.8 (17.3)(20–89)	55.0 (8.1)* (39–77)	45.9 (14.0)* (20–83)	69.8 (13.9) (20–97)	62.0 (9.6) (31–83)	51.2 (16.6) (18–88)
Marital status n (%)							
	Single	4 (7.4)	3 (10.3)	32 (21.3)	17 (5.2)	17 (3.4)	107(10.6)
	Married/Cohabiting	36 (66.7)	22 (75.9)	93 (62.0)	208 (63.4)	434 (86.6)	779 (77.1)
	Widowed/Divorced	13 (24.1)	4 (13.8)	25 (16.7)	103 (31.4)	49 (9.8)	124 (12.3)
	Unknown	1 (1.9)	- (-)	- (-)	- (-)	1 (0.2)	- (-)
Living situation n (%)							
	alone	11 (20.4)	7 (24.1)	39(26.0)	107 (32.7)	73 (14.6)	189 (18.7)
	with partner/child/other	42 (77.8)	22 (75.9)	111 (74.0)	209 (63.9)	427 (85.4)	821 (81.3)
	nursing home	1 (1.9)	- (-)	- (-)	11 (3.3)	- (-)	- (-)
	unknown	- (-)	- (-)	- (-)	1 (0.3)	1 (0.2)	- (-)
Education in years n (%)							
	≤ 6	7 (13.5)	5 (17.2)	17(11.6)	45 (13.7)	44 (8.8)	79 (7.8)
	7–10	18 (34.6)	12 (41.4)	45 (30.8)	90 (27.4)	202 (40.3)	305 (30.2)
	11–16	18 (34.6)	7 (24.1)	33 (22.6)	110 (33.5)	124 (24.8)	260 (25.7)
	≥ 16	9 (17.3)	5 (17.2)	51 (34.9)	77 (23.5)	129 (25.7)	353 (35.0)
	Unknown	2 (3.7)	- (-)	4 (2.7)	6 (1.8)	2 (0.4)	13 (1.3)
PHQ sum score Median (IQR) (min-max)		15.0 (10.0–18.0)(3–27)	7.0 (4.5–12.5)* (1–22)	9.0 (6.0–14.0)* (0–26)	4.0 (2.0–7.0)* (0–23)	1.0 (0.0–4.0) (0–17)	2.0 (1.0–4.0) (0–27)
Type of stroke n (%)							
	Intracerebral hemorrhage	9 (16.7)	- (-)	- (-)	39 (11.9)	- (-)	- (-)
	Infarction	45 (83.3)	- (-)	- (-)	289 (88.1)	- (-)	- (-)
Localization n (%)							
	Right	24 (44.4)	- (-)	- (-)	122 (37.2)	- (-)	- (-)
	Left	21 (38.9)	- (-)	- (-)	142 (43.3)	- (-)	- (-)
	Other	9 (16.7)	- (-)	- (-)	64 (19.5)	- (-)	- (-)
BI 1e wk post-stroke Median (IQR) (min-max)		16.5 (7.0–18.0) (0–20)	- (-) (-)	- (-) (-)	15.0 (9.0–18.0) (0–20)	- (-) (-)	- (-) (-)
BI 6–8 wk post-stroke Median (IQR) (min-max)		19.5 (14.5–20.0) (0–20)	- (-) (-)	- (-) (-)	20.0 (18.0–20.0) (0–20)	- (-) (-)	- (-) (-)

BI, Barthel Index; CIDI, Composite International Diagnostic Interview; SD, standard deviation

* = significant difference in estimate between de depressed versus non-depressed patients of the stroke cohort or between the depressed patients of the stroke cohort versus the depressed patients of the other cohorts;

p <0.05.

In the stroke cohort, 54 patients (14.1%, 95% CI 11.0%-18.0%) were diagnosed with major depressive disorder according to DSM-IV criteria. In the symptomatic atherosclerotic diseases and general practice cohorts, 29 (5.4%, 95% CI 3.8%-7.9%) and 150 patients (12.9%, 95% CI 11.1%-15.0%) were diagnosed with major depressive disorder, respectively. Depression severity, represented by the PHQ sum score, differed between depressed patients in the stroke cohort and the other cohorts. In the stroke cohort, the median PHQ-9 sum score was 15.00 (IQR = 10.00–18.00) compared to a median sum scores of 7.00 (IQR = 4.50–12.50) in the symptomatic atherosclerotic diseases cohort and 9.00 (IQR = 6.00–14.00) in the general practice cohort. These results indicate that stroke patients experience depressive symptoms more frequently than the patients in the symptomatic atherosclerotic diseases cohort and the general practice cohort.

**Fig 1:**
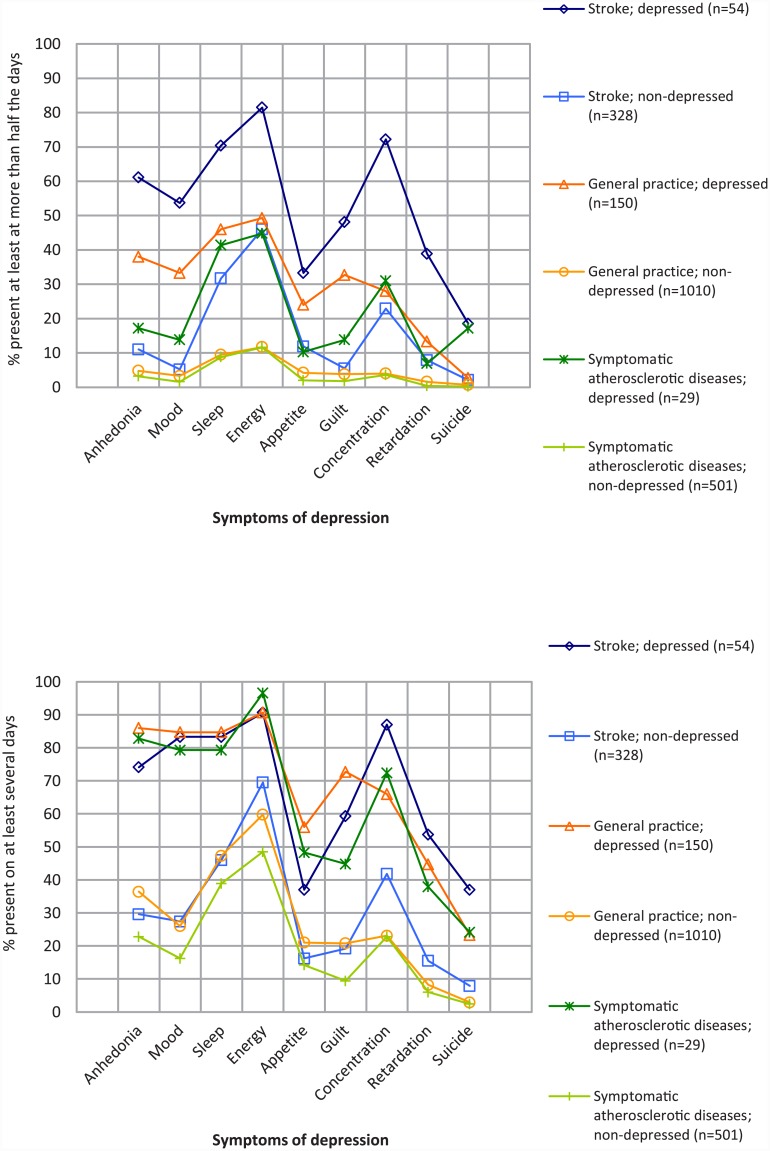
Symptom profiles of stroke patients, patients with other symptomatic atherosclerotic disease and patients in general practice. Symptoms of depression; % present at least at more than half the days. Symptoms of depression; % present on at least several days.

In Fig 1 we depict the symptom profiles of the depressed and non-depressed patients in the three cohorts.

Comparing the symptom profiles of depressed versus non-depressed stroke patients shows that symptom prevalence increased in the depressed stroke patients, including increases in somatic symptoms.

Comparing the symptom profile of depressed stroke patients with that of depressed patients with other symptomatic atherosclerotic diseases other than stroke and the depressed patients in general practice reveals a similar symptom profile pattern in the three cohorts. A notable finding concerns the prevalence of the symptoms in the three cohorts. When we used the original threshold value, indicating a symptom to be present if it occurred at least half of the days [35], the symptom profiles differed somewhat with regard to prevalence.

This difference disappeared when we employed the recently recommended cut-off value when a symptom was considered to be present if it occurred on several days [38]. Using this recently recommended cut-off value, most of the symptoms show an almost equal prevalence in the different patient populations.

Age and sex slightly biased these findings; the odds ratio of the prevalence of the symptoms ‘little interest or pleasure in doing things and ‘moving or speaking slowly or being fidgety or restless’ are reduced by including age and sex in the regression model, and the odds ratios of the prevalence of the symptom ‘trouble falling or staying asleep or sleeping too much’ are increased by including age and sex in the regression model (Table 2). Small differences between the prevalence of depressed patients with other symptomatic atherosclerotic diseases than stroke and the depressed patients in general practice were shown in the symptoms ‘little interest or pleasure in doing things’, ‘poor appetite or overeating’, and ‘trouble concentrating’. In multivariable regression analyses the difference between the prevalence of the symptoms ‘feeling bad about himself’ and ‘trouble concentrating’ are explained by the patient population, whereas the prevalence of the symptoms ‘poor appetite or overeating’ and ‘moving or speaking slowly or being fidgety or restless’ are explained by age, but not by patient population.

**Table 2:** Multivariable logistic regression of symptoms of depression with age, sex and population as indicator.

Outcome		Predictors Population General practice^a^		Predictors Population Symptomatic atherosclerotic disease^b^	
		Crude OR (95% CI)	Adjusted^b^ OR (95% CI)	Crude OR (95% CI)	Adjusted^b^ OR (95% CI)
**Symptoms of Depression** present at more than half the days					
	Anhedonia	0.39 (0.21–0.74)	0.47 (0.22–0.97)	0.13 (0.04–0.40)	0.16 (0.05–0.49)
	Mood	0.43 (0.23–0.81)	0.47 (0.22–0.97)	0.14 (0.04–0.45)	0.16 (0.05–0.53)
	Sleep	0.36 (0.18–0.70)	0.24 (0.11–0.53)	0.30 (0.12–0.76)	0.26 (0.10–0.72)
	Energy	0.22 (0.10–0.47)	0.21 (0.09–0.50)	0.19 (0.07–0.50)	0.20 (0.07–0.58)
	Appetite	0.63 (0.32–1.25)	0.64 (0.29–1.41)	0.23 (0.06–0.87)	0.23 (0.06–0.91)
	Guilt	0.52 (0.28–0.98)	0.52 (0.25–1.09)	0.17 (0.05–0.56)	0.20 (0.06–0.66)
	Concentration	0.15 (0.08–0.30)	0.15 (0.07–0.34)	0.17 (0.07–0.46)	0.17 (0.06–0.48)
	Retardation	0.24 (0.12–0.50)	0.37 (0.16–0.86)	0.12 (0.03–0.54)	0.17 (0.04–0.84)
	Suicide	0.12 (0.04–0.40)	0.13 (0.03–0.52)	0.92 (0.28–2.99)	1.2 (0.31–4.39)
**Symptoms of Depression** present at several days					
	Anhedonia	2.15 (1.00–4.61)	3.05 (1.23–7.56)	1.68 (0.54–5.25)	2.13 (0.64–7.08)
	Mood	1.10 (0.48–2.56)	1.42 (0.54–3.73)	0.77 (0.24–2.42)	0.85 (0.26–2.86)
	Sleep	1.10 (0.48–2.56)	0.97 (0.36–2.58)	0.77 (0.24–2.42)	0.60 (0.18–2.04)
	Energy	0.99 (0.34–2.90)	1.01 (0.29–3.50)	2.86 (0.32–25.70)^c^	2.82 (0.30–26.78)^c^
	Appetite	2.16 (1.14–4.10)	1.59 (0.76–3.30)	1.59 (0.64–3.96)	1.34 (0.51–3.50)
	Guilt	1.83 (0.95–3.50)	1.60(0.75–3.41)	0.56 (0.23–1.39)	0.52 (0.20–1.37)
	Concentration	0.29 (0.12–0.69)	0.25 (0.09–0.65)	0.39 (0.13–1.22)	0.34 (0.10–1.10)
	Retardation	0.70 (0.37–1.30)	0.99 (0.48–2.06)	0.53 (0.21–1.32)	0.55 (0.21–1.46)
	Suicide	0.52 (0.27–1.01)	0.50 (0.23–1.09)	0.54 (0.20–1.49)	0.51 (0.18–1.48)

The logistic regression is conducted in the depressed patients of the three cohorts. The number of depressed patients in each cohort were in the stroke cohort n = 54, in the general practice cohort n = 150, and in the symptomatic atherosclerotic disease cohort n = 29.

OR, odds ratio; CI, Confidence Interval

^a^ reference category is ‘Stroke’

^b^ The odds ratios are adjusted for Age and Sex, using multivariable logistic regression.

^c^ The broad confidence intervals are the result of the very high prevalence of this symptom in the patients with symptomatic atherosclerotic disease other than stroke.

This indicates that the symptom profiles are quite similar in depressed stroke patients compared with depressed patients with other symptomatic atherosclerotic diseases and depressed patients in general practice. However, in depressed stroke patients the severity of the depressive symptoms is higher than in depressed patients in general practice and those with symptomatic atherosclerotic diseases.

## Discussion

We compared the clinical manifestation of the symptoms of major depressive disorder in stroke patients with that in patients with other symptomatic atherosclerotic diseases and patients in general practice. Our findings suggest that both somatic and non-somatic symptoms in patients with depression showed a broadly similar profile pattern, irrespective of whether they occur post stroke. However, the severity of symptoms is higher in stroke patients.

To appreciate these findings, some aspects of the study need to be addressed. To our knowledge, this is the first study in which the symptom profile of depression in stroke patients is compared with two other patient populations. Most of the prior studies only compared depressed stroke patients with non-depressed stroke patients [15–18], patients with endogenous depression [19–22], or other somatic patient populations, such as those with myocardial infarction or geriatric patients [23–25]. Moreover, we used large cohorts, conducted the same diagnostic interview and used the same depression screening instrument in all of the three cohorts. These instruments were shown to be valid in the different patient populations [34,36–38].

A limitation of the study is that we only included stroke patients who were able to communicate adequately because assessing depression with the PHQ-9 and CIDI is highly dependent on verbal and cognitive competence. The difficulty associated with reliably measuring depression in patients with cognitive and communicative disorders limits the generalizability of our results to patients who are able to communicate [39]. In our stroke cohort, the prevalence of depression after stroke was 14.1%. Although the literature describes considerable variation in the frequency of depression after stroke, a pooled estimate indicates that depressive symptoms are present in one-third of all stroke survivors at any given time during follow-up [1]. The prevalence of depression after stroke in our study could have been influenced by the follow up of eight weeks or the exclusion of patients who were too ill to participate due to stroke severity or severe cognitive or communicative impairments. Previous studies have shown a correlation between depression and stroke severity, as well as cognitive impairment and depression [4,10,39]. The stroke patients were generally older than those with other symptomatic atherosclerotic diseases and general practice patients. Additionally, there were more male patients in the symptomatic atherosclerotic diseases cohort, whereas the proportions of male and female patients in the stroke cohort were equal. Although these differences biased the results slightly, this does not change the finding that depressive symptoms in patients with depression in the three cohorts showed a broadly similar profile pattern, given the slight amount of change in the odds ratios. Finally, we did not register medication use in the stroke cohort and were therefore not able to account for the potential effects of antidepressants. This limitation could have affected our results because medication use influences the degree to which patients experience depressive symptoms, which affects symptom prevalence.

Considering our results in relation to other studies, we identified some interesting findings. First, all of the symptoms, including the somatic symptoms, were more prevalent in the depressed stroke patients compared with the non-depressed stroke patients. This finding is consistent with previous studies that have concluded that all depressive symptoms are more frequent in depressed stroke patients [15–18]. This is interesting since the prevailing view of health care professionals is that somatic symptoms are the consequence of the stroke more than the clinical manifestation of depression after stroke, which suggests that somatic symptoms are equally prevalent in depressed as in non-depressed stroke patients. Our findings, however, do not support this view, but strengthen the evidence that somatic symptoms should also be interpreted as a manifestation of depression. Second, the symptom profile was not different in the depressed stroke patients from the symptom profile in patients with other symptomatic atherosclerotic diseases and general practice patients. This differs from other studies showing that symptom profiles differ between patient populations [21–23,26]. However, our study differs from others in that we investigated the symptom profile in more detail. This could explain the differences with previous studies, which is illustrated by the following: using the original threshold value ‘symptoms present more than half the days’ suggests that there are differences between the stroke patients and the other patient populations, which supports the findings in the literature that symptom profiles differ between patient populations [21–23,25, 26]. However, using the reset cut-off value ‘symptoms present on at least several days’ indicates that there is no difference in symptom profile, which corroborates findings in other studies [20,24]. Hence, the comparison of prevalence rates at the different cut-off value allows us to conclude that depressed stroke patients have a similar symptom profile, but they suffer from more severe symptoms than depressed patients with other symptomatic atherosclerotic diseases or general practice depressed patients.

In this study, we focused on the clinical manifestation of depression after stroke. Our findings are of importance for clinical practice, directing health care professionals in the identification of depression after stroke. A conclusive explanation of the nature and origin of depression after a stroke, however, cannot be given based on these data. Therefore, further research is needed to better understand the direct role of biological factors provoked by the brain injury and vascular pathophysiology underlying the stroke, such as perfusion deficits and inflammation as well as the psychological response to the physical, cognitive, and social impairments produced by the stroke [7,8,40].

In conclusion, we found that the clinical manifestation of depression after stroke does not differ from depression in other patient populations; the symptom profile patterns were similar in depressed stroke patients compared with depressed patients with other symptomatic atherosclerotic diseases, as well as with depressed patients in general practice. Our results suggest that the somatic symptoms in the depressed stroke patients should be considered as clinical manifestation of depression after stroke. Furthermore, the high prevalence of depressive symptoms in the early stage after stroke highlights the importance of early detection of depression after stroke. Provided that early detection is followed by treatment and follow-up, taking into account the advantages and disadvantages of different treatment options [12,41,42], this early detection may decrease the burden of depression after stroke and the negative impact that depression has on stroke patient participation in rehabilitation.
